# Detection of Circulating Tumor Cells and Microbial DNA Fragments in Stage III Colorectal Cancer Patients under Three versus Six Months of Adjuvant Treatment

**DOI:** 10.3390/cancers13143552

**Published:** 2021-07-15

**Authors:** Asimina Koulouridi, Ippokratis Messaritakis, Emmanouil Theodorakis, Maria Chondrozoumaki, Maria Sfakianaki, Nikolaos Gouvas, John Tsiaoussis, Dimitrios Mavroudis, Maria Tzardi, John Souglakos

**Affiliations:** 1 Laboratory of Translational Oncology, Medical School, University of Crete, 70013 Heraklion, Greece; medp2011871@med.uoc.gr (A.K.); bio2885@edu.biology.uoc.gr (E.T.); med3425@edu.med.uoc.gr (M.C.); msfakianak@uoc.gr (M.S.); mavroudis@uoc.gr (D.M.); souglak@uoc.gr (J.S.); 2 Department of Medical Oncology, University General Hospital of Heraklion, 70013 Heraklion, Greece; 3 Medical School, University of Cyprus, 20537 Nicosia, Cyprus; gouvas.nikolaos@ucy.ac.cy; 4 Department of Anatomy, School of Medicine, University of Crete, 70013 Heraklion, Greece; tsiaoussis@uoc.gr; 5 Laboratory of Pathology, Medical School, University of Crete, 70013 Heraklion, Greece; tzardi@med.uoc.gr

**Keywords:** colorectal cancer, stage III, adjuvant chemotherapy, circulating tumor cells, microbial DNA, three vs. six months adjuvant treatment

## Abstract

**Simple Summary:**

Duration of adjuvant therapy in stage III CRC is now under re-evaluation. The aim of the current study was the detection of microbial DNA that could be originated from the intestine (16S rRNA, *E. coli*, *B. fragilis* and *C. albicans*) and the detection of CTCs during treatment with FOLFOX or CAPOX, in stage III CRC patients. CTCs were significantly decreased after 3 months of treatment, whereas 6 months resulted to their increase again. A significant increase of CTCs was demonstrated in patients under FOLFOX for 6 months. A significant correlation was demonstrated following microbial DNAs and both CTCs detection at baseline and CTCs increase, between baseline and 3 months of treatment. The results provide additional evidence of non-inferiority of 3 over 6 months of treatment, mainly in patients under CAPOX.

**Abstract:**

Oxaliplatin-fluoropyrimidine combination therapy is the gold standard treatment for patients with stage III colorectal cancer (CRC); however, treatment duration is now under re-evaluation. The aim of the study was the evaluation of the non-inferiority of three over six months treatment with FOLFOX or CAPOX, in stage III CRC patients. Peripheral blood samples from 121 patients were collected, at three time points during treatment and evaluated for circulating tumor cells (CTCs) and microbial DNA detection (16S rRNA, *Escherichia coli*, *Bacteroides fragilis*, *Candida albicans*). Of all patients, 41.3% and 58.7% were treated with FOLFOX and CAPOX, respectively. CTCs were significantly decreased and increased after three and six months of treatment, respectively. CAPOX tends to reduce the CTCs after 3 months, whereas there is a statistically significant increase of CTCs in patients under FOLFOX after 6 months. A significant correlation was demonstrated between microbial DNA detection and both CTCs detection at baseline and CTCs increase between baseline and three months of treatment. To conclude, the current study provides additional evidence of non-inferiority of three over 6 months of treatment, mainly in patients under CAPOX.

## 1. Introduction

Colorectal cancer (CRC) remains the third most frequent cancer type worldwide and a common reason of mortality because of solid tumors [1]. Stage III CRC patients have up to 60% 5-years overall survival (OS) and adjuvant chemotherapy aims to raise this percentage and prolong both the OS and the disease-free survival (DFS) [2]. The oxaliplatin- fluoropyrimidine combination chemotherapy, Fluorouracil-leucovorin-oxaliplatin (FOLFOX) or capecitabine-oxaliplatin (CAPOX) for 6 months was the gold standard treatment for such a group of patients [2,3]. However, oxaliplatin may lead to adverse effects, and especially neuropathy [4]. Usually, neuropathy starts soon after the regimen infusion and it takes few days to resolve. Sometimes, peripheral neuropathy can remain for longer periods (up to 3–5 years) lowering the patients’ quality of life [5]. Moreover, the total cost of adjuvant therapy for stage III CRC patients in Europe would be reduced for a half a billion by using three months of CAPOX instead of 6 months of FOLFOX [5]. IDEA collaboration was an attempt to reduce the time of adjuvant treatment [6]. The primary endpoint of IDEA was the non-inferiority of using three versus 6 months of adjuvant treatment, by comparing 3-year DFS. In a total of 12,834 patients who received three months or 6 months of adjuvant chemotherapy, the non-inferiority statistical margin was not reached. However, it was shown that the use of CAPOX for three months, especially in low-risk patients could be as effective as 6 months treatment [6,7]. However, concerning OS, despite the non-inferiority of three months of adjuvant treatment was not achieved, the difference of 0.4% that there was in 5-year OS between the two different treatment durations, should be under clinical evaluation [8].

Liquid biopsy is playing a new, crucial role on the detection and monitoring of different cancer types. Its role is important for early diagnosis, treatment selection, as a prognostic tool or for monitoring treatment efficacy [9]. Circulating tumor cells (CTCs) escape to bloodstream from the tumor and are implicated in the procedure of micrometastasis [10]. Various technologies have been used for their detection: immunocytological, molecular and functional assays [11]. It has been shown that CTCs have a prognostic and predictive value for patients with CRC [12,13,14,15].

Carcinoembryonic antigen (CEA) is the most frequently used marker for the recognition of tumors cells in CRC. Immunohistochemistry has been used CEA, for years as a characteristic of CRC tumors, and has been validated through this time [16,17]. Molecular methods have also been developed. Our group has previously developed such a reproducible method for the detection of CEA-like cellular adhesion molecule 5 (*CEACAM5*) mRNA positive CTCs in patients with CRC [12,18], and such a detection revealed their prognostic value [12].

Gut microbiota seems to play an important role to human health. The intestinal dysbiosis, is linked to various immune and metabolic conditions [19]. Dysbiosis is one of the mechanisms that lead to microbial translocation, which refers to the passage of intestinal microbes into the bloodstream. Not only these microbes, but also their products (lipopeptides, endotoxins, peptidoglycan and nucleid acids, could be detected in the blood [20]. These microbial fragments can be detected by several molecular techniques [21,22,23,24,25]. As it has been reported previously, the microbial DNA detection in the blood of such patients highlights the involvement of such microbes in CRC tumorigenesis, disease progression/recurrence, and overall survival [21,22,23,24,25].

With this prospective, single institution study, we aimed to the detection and comparison of CEACAM5-positive CTCs with microbial DNA detection which could be originated from the intestine, in the blood of patients with stage III CRC under three versus 6 months of chemotherapy. To our knowledge, this is the first time that laboratory parameters are analyzed and compared in an attempt to prove non-inferiority of three vs 6 months of adjuvant treatment.

## 2. Materials and Methods

### 2.1. Patients Enrollment and Ethics Approval

In total, 121 patients with stage III CRC were included in the present, single-centered study. Inclusion criteria involved the age >18 years old of the enrolled patients, presence of stage III CRC adenocarcinoma following curative surgical resection and adjuvant therapy initiation with FOLFOX or CAPOX within 8 weeks following surgery. Patients with a presence of additional solid tumors were excluded from the study. All patients enrolled are patients of the Department of Medical Oncology, University Hospital of Heraklion.

### 2.2. Blood Sampling

Peripheral blood (15 mL in EDTA) was collected at the middle-of-vein puncture. To avoid contamination with skin epithelial cells, the first 5 mL of blood were discarded.

Peripheral blood mononuclear cells (PBMCs) were isolated by Ficoll–Hypaque (d = 1077 g/mL; Sigma-Aldrich, GmbH, Taufkirchen, Germany) gradient density centrifugation at 1800 rpm, for 30 min. Slide cytospins, RNA extraction and genomic DNA isolation were prepared as previously described [12,14,25,26,27].

### 2.3. Double Immunofluorescence Assay (IFAT)

Cells that express CEACAM5 but not CD45 which recognizes hematopoietic cells (CEACAM5+/CD45-) where considered as positive CTCs. CEACAM5 was detected using the FITC conjugated monoclonal antibody against CEACAM5 (anti-mouse: Abcam, Cambridge, UK) and CD45 (anti-rabbit: Common Leukocyte Antigen; Santa Cruz, CA, USA) was labelled with Alexa 555 (Molecular Probes, Invitrogen, Rockford, IL, USA). In brief, aliquots of 1 × 10^6^ PBMCs were cytocentrifuged on microscope slides (at 2000 rpm for 2 min). Cytospins were air dried and stored at −80 °C, until use. One slide per patient was analyzed at each time point. Prior staining, PBMCs were fixed for 20 min, in ice-cold acetone:methanol 9:1 (*v*/*v*). Incubation time was 1 h for all antibodies. Additionally, DAPI-antifade (Molecular Probes) was added to each sample to obtain nuclear staining. Slides were analyzed under a fluorescence microscope (Leica DM 2500, Heidelberg, Germany). Results are expressed as number of CTCs/10^6^ PBMCs.

### 2.4. Reverse Transcription-Quantitative PCR (RT-qPCR)

The reverse transcription and the qPCR conditions were performed as previously described [12,14]. In brief, the NanoDrop (Thermo Fisher Scientific, Wilmington, DE, USA) equipment was used to measure RNA concentration. *β*-actin gene amplification was used for RNA integrity verification. RNA from the Lovo (colorectal cell line) and ARH-77 (leukemic cell line) were used as positive and negative controls, respectively. ABI Prism 7900HT Sequence Detection System (Applied Biosystems, Waltham, Massachusetts, USA) was used for gene expression quantification. All experimental analysis run in triplicates. An external calibration curve obtained using external standard cDNAs was used for quantification [12,14]. In brief, RNA from 1 × 10^6^ Lovo cells was used for cDNA synthesis of Lovo cells RNA serial dilutions (1–10^5^) and was analyzed in each run. By plotting the number of Lovo cells that correspond to each external standard cDNA vs the value of its quantification cycle, the calibration curve was created. The number of circulating *CEACAM5*mRNA+cells for all patients’ samples was expressed as cell equivalents/5 μg of total RNA, based on the external standard calibration curve. The limit of detection (LOD) of the assay was found to correspond to 0.7 Lovo cell equivalents/5 μg of RNA (LOD = 3.3 SD/slope, where SD is the standard deviation of the quantification cycle for 1 Lovo cell equivalent) [12]. Analysis was performed using the SDS 2.3 software.

### 2.5. Microbial DNA Amplification by PCR

DNA isolation from the whole blood collected before the initiation of adjuvant treatment was done with the QIAamp DNA Blood Mini kit (QIAGEN, Hilden, Germany) following the manufacturer’s instructions. The NanoDrop ND-1000 v3.3 (Thermo Fisher Scientific, Rockford, IL, USA) equipment was used for DNA quantification. All materials and conditions for each gene target involved in the present study have been previously described by our group [25]. In brief, three primer pairs were used to detect bacterial genomic DNA encoding 16S rRNA; glutamine synthase of *Bacteroides fragilis*; *β*-galactosidase gene of most *Escherichia coli* and one primer pair to detect 5.8S rRNA found in *Candida albicans*; the human glyceraldehyde phospho-dehydrogenase (GAPDH) was used as a reference gene to verify DNA integrity of the samples. 16S rRNA was used as a reference in the detection of bacterial (only) DNA in the blood samples.

### 2.6. Study Design and Statistical Analysis

The current study is a prospective study, investigating the detection of CEACAM5 and *CEACAM5*mRNA in CTCs and of microbial DNA fragments in the blood of CRC patients. The experiments and the evaluation of results were done blindly to patients’ data. Statistical analysis was done under the SPSS v. 26 environment (IBM Corp. Armonk, New York, USA, as previously described [28]. Statistical significance was set by the user at *p* = 0.05.

## 3. Results

### 3.1. Patient’s Characteristics

The patients’ characteristics are listed in Table 1. In brief, 121 patients were included in the study. Patients median age was 62 years (range: 37–83 years), 73 (60.3%) were males, 112 (92.6%) had a colon/sigmoid tumor location, 28 (23.1%) had tumors of the right colon, 99 (85.3%) were diagnosed with adenocarcinoma and 71 (58.7%) received CAPOX. Moreover, 9 (7.4%) and 41 (33.9%) patients were enrolled in the FOLFOX regimen for 3 and 6 months respectively, whereas 28 (23.1%) and 43 (35.6%) patients were enrolled in the CAPOX regimen for 3 and 6 months respectively. For patients enrolled in the 6 months treatment administration, CTC counts are also available at three months, during treatment. PS-ECOG (Performance Status according to the Eastern Cooperative Oncology Group) for all patients, but one, 0–1. Moreover, in total 29 (24%) patients relapsed (Table 1 and Table S1) following treatment completion, 17 (58.6%) of which treated for 6 months (Table 1 and Table S1).

### 3.2. Detection of CTCs

#### 3.2.1. Double Immunofluorescence Assay (IFAT)

**Figure 1:**
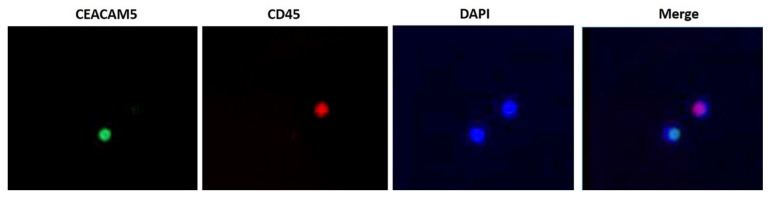
Representative figures taken after double immunofluorescence staining (x40 magnification). As a circulating tumor cell (CTC) was considered a cell that is CEACAM5-positive and CD45-negative. Two cells are presented here: A CTC which expresses CEACAM5 (FITC-green) but not CD45 and a hemopoietic cell which expresses CD45 (Alexa 555-red) but not CEACAM5.

Table 2 and Table S1 demonstrate the results regarding the CTCs detection using IFAT, at all three time points. At the time of the analysis, not all patients have completed their 3- or 6-months treatment administration. Hence, in total 121 samples were available at baseline; 69 patients at 3 months and 54 patients at 6 months. Only CEACAM5+/CD45- cells were considered as CTCs (Figure 1). As it was observed, positivity was almost at the same level, for all three time points (baseline: 45%; 3 months: 44.9%; 6 months: 44.4%) (Table 2 and Table S1). However, between baseline and three months of treatment, the absolute number of CTCs was significantly decreased [median: 3 (range: 1–119) vs. median: 1 (range: 1–6); *p* < 0.001) (Table 2 and Table S1). Additionally, a significant increase was observed between 3 and 6 months of treatment, in the absolute number of CTCs [median: 1 (range: 1–6) vs. median: 2 (range: 1–134); *p* < 0.001) (Table 2 and Table S1).

Moreover, the changes in the detection of CTCs were evaluated during treatment. As it was observed, of those patients with CEACAM5-positive cells at baseline, 27.5% had detectable CEACAM5-positive cells after three months of treatment, whereas 20.3% of patients eliminated their CTCs (Table 3 and Table S1). Of those patients with undetectable CTCs, 33.3% and 18.8% remained negative or became positive, respectively (Table 3 and Table S1). Between baseline and 6 months of treatment, 22.2%, 5.6%, 50% and 22.2% remained positive, eliminated their CTCs, remained negative or became positive, respectively (Table 3 and Table S1). There was a significantly greater elimination of CTCs in patients under 3 vs 6 months of treatment (*p* = 0.044, Table 3). Moreover, as it was demonstrated, 57.1%, 14.3% 14.3% and 14.3% of the patients had detectable CTCs both at 3 and 6 months of treatment (remained positive), had detectable CTCs at 3 months but no CTCs were detected at 6 months of treatment (eliminated their cells), had undetectable CTCs at both time-points (remained negative) or had undetectable CTCs at 3 months but detectable CTCs at 6 months of treatment (became positive) (Table 3 and Table S1).

In the means of the absolute number of CTCs, 23.2% and 37.7% of the patients increased and decreased their CTCs, respectively, whereas in 39.1% of the patients, the number of their CTCs remained stable (0 or 1 cell), at both time-points. (Table 3 and Table S1). Between baseline and 6 months, the number of CTCs increased, decreased, or remained stable in 29.6%, 14.8% and 55.6% patients, respectively (Table 3 and Table S1). Finally, between 3 and 6 months of treatment, the number of CTCs increased, decreased, or remained stable in 42.9%, 23.8% and 33.3% of the patients, respectively (Table 3 and Table S1). As it was observed, 3 out of 6 months of treatment led to a decrease in the absolute number of CEACAM5-positive cells in a higher percentage of patients (37.7% vs 14.8%, respectively), whereas between 3 and 6 months resulted to significant increase in 42.9% of patients (*p* = 0.007; Table 3 and Table S1).

Furthermore, CTC detection at baseline was more common in patients under 70s than those ≥70 years old (44 vs. 11 patients; *p* = 0.006). However, a higher number of patients ≥70 and a lower number of <70 years old was demonstrated to decrease their CTCs between baseline and 3 months of treatment (22 vs. 4 patients; *p* = 0.033) (Table S1). No other correlations were observed between patients’ characteristics and CTC detection.

#### 3.2.2. Reverse Transcription—Quantitative PCR

From the analysis of RT-qPCR results, it was observed that both the positivity of patients and CTCs copy numbers reduced between baseline and three months of treatment [38.3%; median copy numbers: 1.1 (range: 0.71–6.7) vs. 34.8%; median copy numbers: 1.1 (range: 0.75–2.4]; whereas, an increase was observed again, after 6 months of treatment [42.6%; median copy numbers: 1.2 (range: 0.71–12.33] (Table 2 and Table S1). It is worth mentioning that there was a statistically significant correlation of the results obtained between IFAT and RT-qPCR, at all time points (*p* > 0.001; *p* > 0.001 and *p* > 0.001, respectively; Table 2).

#### 3.2.3. Association of Chemotherapy Regimen with Changes in the Absolute Number and Copy Number of CTCs during Treatment

By associating the regimen administered with the absolute number of CTCs detected by IFAT, it was revealed that patients under CAPOX decreased their detectable CTCs after three months, despite of not statistical significance (*p* = 0.554; Table 4 and Table S1). In the case of patients under FOLFOX, significantly increased CTCs were observed after administration of chemotherapy for 6 months (*p* = 0.001; Table 4 and Table S1). A significantly increased number of detectable CTCs was also observed between 3 and 6 months in patients under FOLFOX (*p* = 0.05; Table 4 and Table S1). Similarly, by associating the regimen administered with the copy numbers of CTCs detected by RT-qPCR, a significant increase in copy numbers was observed between baseline and 6 months (*p* = 0.011) and also between three and 6 months (*p* = 0.046) in patients under FOLFOX (Table S1).

**Figure 2:**
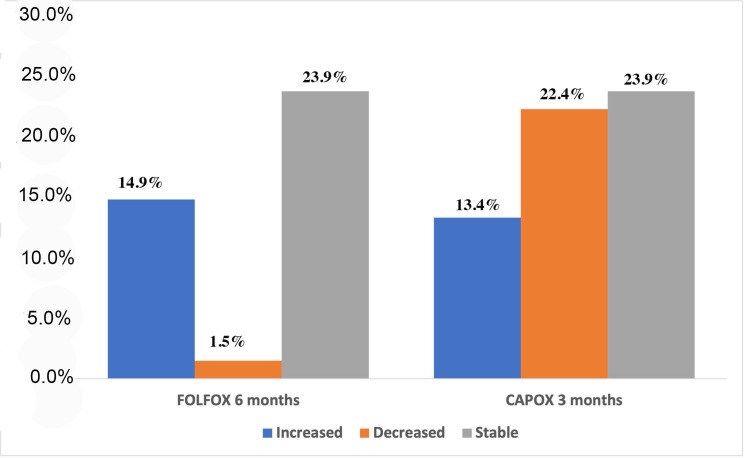
Rates (%) of patients whose CTC number increased, decreased or remained stable during different treatment regimens (FOLFOX for 6 months vs CAPOX for 3 months).

Furthermore, the association of the absolute CTCs detection number was investigated in patients under CAPOX for 3 months vs FOLFOX for 6 months. As it was demonstrated, 30.3% and 17% of the patients under CAPOX for 3 and FOLFOX for 6 months, respectively, had detectable CTCs. This was not of any statistical significance (*p* = 0.9), thus providing additional evidence of non-inferiority of 3 out of 6 months of treatment (Table S1). Additionally, as demonstrated in Figure 2, 10 (14.9%) and 9 (13.4%) patients under FOLFOX for 6 and CAPOX for 3 months, respectively, increased the number of their CTCs in their blood; whereas 1 (1.5%) and 15 (22.4%) patients under FOLFOX for 6 and CAPOX for 3 months, respectively, decreased their CTCs, and this is of a statistical significance (*p* = 0.004) (Figure 2 and Table S1).

### 3.3. Detection of Microbial DNA Fragments

**Figure 3:**
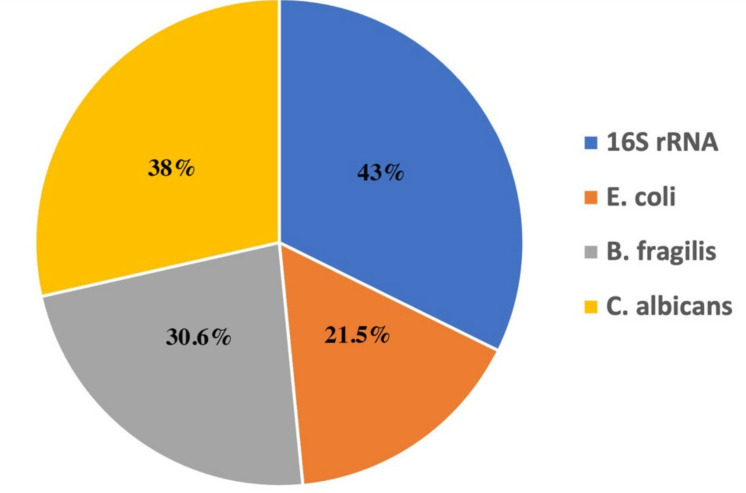
Detection rates (%) of microbial DNA at baseline in stage III CRC patients.

Detection of microbial DNA for 16S rRNA, *E. coli*, *B. fragilis* and 5.8S rRNA of *C. albicans* was detected in the blood of 52 (43%), 26 (21.5%), 37 (30.6%) and 46 (38%) stage III CRC patients at baseline (Figure 3 and Table S1).

A significant association was demonstrated with the CTC detection at baseline and all four microbial DNAs detected (*p* = 0.026, *p* = 0.02, *p* < 0.001 and *p* = 0.029 for 16S rRNA *E. coli*, *B. fragilis* and 5.8S rRNA of *C. albicans*, respectively; Table 5) when all patients were evaluated. Moreover, a statistically significant association was revealed between the detection of microbial DNA coding for 16S rRNA and glutamine synthase gene of *B. fragilis* with the increase of CTCs number between baseline and three months of chemotherapy when all patients were evaluated (Table 5). When patients were grouped according to treatment regimen, it was demonstrated that patient under FOLFOX presented a significant association between baseline CTCs detection and *E. coli* (*p* = 0.036), baseline CTCs detection and *B. fragilis* (*p* = 0.01) and between the detection of microbial DNA coding for 16S rRNA and the increase of CTCs number between baseline and three months of chemotherapy (*p* = 0.034) (Table 5). Moreover, in patients under CAPOX, a significant association was demonstrated between baseline CTCs detection and *B. fragilis* (*p* = 0.015), baseline CTCs detection and 5.8S rRNA of *C. albicans* (*p* = 0.029), 3 months CTCs detection and 16S rRNA (*p* = 0.043), 3 months CTCs detection and *B. fragilis* (*p* = 0.013), and between the detection of microbial DNA coding for 16S rRNA or *B. fragilis* and the increase of CTCs number between baseline and 3 months of chemotherapy (*p* = 0.009 and *p =* 0.015, respectively) (Table 5). No other significant correlations were demonstrated with microbial DNA detection and patients’ characteristics at any treatment regimen.

## 4. Discussion

CRC, at a large extent is preventable and curable disease [29]. Even, for stage III CRC adjuvant chemotherapy (FOLFOX or CAPOX) reduced disease recurrence risk and mortality by 30% and 32%, respectively [2,3,30,31,32]. The gold standard use of oxaliplatin-based regimens for 6 months is usually accompanied with several adverse events. Neuropathy is the main problem for a number of patients. Neuropathy can be long lasting and have an impact on the patients’ quality of life, often leading to treatment discontinuation [33]. The IDEA pooled analysis of six randomized trials, was the first attempt aiming to shorten the duration of adjuvant treatment [6]. The study did not achieve the expected statistical significance for shortening adjuvant treatment duration from 6 to 3 months, in CRC patients; however, there was a clear trend for patients with lower risk CRC (T1-T3/N1) to benefit from the administration of CAPOX for three months [6]. Although the interpretation of the IDEA’s results was hard, it was revealed that tumor’s special characteristics and patients’ desires about quality of life may define regimen and treatment duration [5].

Liquid biopsy is a promising tool for tumor characterization [34] and CTCs detection in CRC patients has a prognostic and predictive value [12,14,15,35,36,37]. The aim of the study was to evaluate the detection and number of CTCs at baseline, after 3 (for all patients) and after 6 months of treatment administration (for patients under 6 months treatment) and correlate such evaluation with microbial DNA fragments in the blood of stage III CRC patients. Gut microbiota has been shown to be implicated in tumorigenesis and disease outcome. Microbial DNA fragments detection in patients with stage II/III or IV has already been demonstrated by our group, earlier [25].

To our knowledge, this is the first laboratory approach to evaluate the effect of 3 versus 6 months of adjuvant treatment, in stage III CRC patients. As it was shown, the rate of patients with detectable CTCs was nearly the same at all time points evaluated (IFAT: 44.4–45% and RT-qPCR: 34.8–42.6%); The results of our study are in agreement with Sotelo et al., who demonstrated approximately the same positivity rates (IIIA: 40%, IIIB: 32%, IIIC: 47%), despite the use of other detection methodologies [38]. Hoon Baek et al., demonstrated that a higher rate (84.1%) of patients had detectable CTCs, preoperatively [39]. Dizdar et al., shown that 31.3–41.3% CRC patients with no detectable metastases or with hepatic metastases, had detectable CTCs postoperatively, depending on methodology [40]. Recently, Hendricks et al., studied the rate of patients with detectable CTCs with three different methods: NYONE^R^ cell imager, ScreenCell^R^ and PCR [41]. The rates of positivity were: 36.4%, 100% and 80%, respectively [41]. In the current study, significant changes were observed in the absolute number of CTCs. To this end, median number and range of CTCs presented a significant decrease after three months of adjuvant treatment, especially in patients under CAPOX. A significant increase in the absolute number of CTCs was demonstrated, especially in patients under FOLFOX. Therefore, both IFAT and RT-qPCR, revealed that 3 out of 6 months administration presented higher treatment efficacy. By a literature review, only studies on metastatic CRC and changes in positivity rate of CTCs during the treatment were demonstrated. Cohen et al., aimed to demonstrate the prognostic and predictive value of CTCs in patients with metastatic CRC. The authors showed that the number of patients with detectable CTCs decreases during treatment (from baseline to twenty weeks) [13]. Bidard et al., showed that in patients with potentially respectable metastatic CRC, there was an elimination of CTCs during preoperative treatment until resection (19% at baseline, 3% at week 4 and 0% before surgery) [42].

Overall, it is reasonable to hypothesize that patients under adjuvant chemotherapy, longer than 3 months, might acquire resistant CEACAM5-positive CTC clones, leading to their accumulation after 6 months of treatment. However, to prove this hypothesis, extensive research is needed. Moreover, regarding microbial DNA fragments, the results of the present study, in stage III CRC patients are in agreement with the results presented earlier by Messaritakis et al., in early CRC patients (stage II/III) [25]. In brief, 43%, 21.5%, 30.6% and 38% of stage III CRC patients had detectable microbial DNA for 16S rRNA, *E. coli*, *B. fragilis*, and 5.8S rRNA in *C. albicans*, respectively. Messaritakis et al., demonstrated that 42.8%, 21.2%, 31.2% and 37% of stage II/III CRC patients had detectable microbial DNA for 16S rRNA, *E. coli*, *B. fragilis*, and 5.8S rRNA in *C. albicans*, respectively. These rates were increased to 88.4%, 31.7%, 82% and 81% respectively, in stage IV CRC patients [25].

Finally, there was a significant correlation between the detection of all microbial DNAs evaluated and the detection of CEACAM5-positive cells at baseline, whereas a significant correlation was revealed between the detection of microbial DNA encoding for 16S rRNA and glutamine synthase gene of *B. fragilis,* and the increase in CTCs after three months of treatment. A possible explanation of such correlation might be the delayed treatment response due to the presence of bacterial species [43,44,45,46]. As mentioned earlier, no statistical significance was demonstrated among the detection of any microbial DNA and the increase in CTC from baseline to 6 months, or from 3 to 6 months. A possible scenario for this might be again the acquired chemoresistance, which might determine treatment response beyond the period 3 months, thus displacing other factors (such as microbial components).

Extensive research is needed to evaluate whether such microbial DNA fragment detection which is possibly originated from the intestine was implicated in the treatment efficacy, or if potential intervention to microbial synthesis (remodeling) might boost treatment. On the other hand, the present is a non-randomized prospective study and carries all the limitations of a randomized trial and of a relatively small size study. For these reasons, the results should be interpreted with caution, and mainly as hypothesis generated. Further studies, longer patients’ follow up and correlations with clinico-pathological features are needed, aiming to a personalized treatment decision, in the means of regimen and duration.

## 5. Conclusions

In conclusion, our study is the first laboratory attempt to compare two different oxaliplatin-based regimens administered as 3- or 6-months adjuvant chemotherapy, in stage III CRC patients. The detection of CTCs using IFAT is significantly correlated with RT-qPCR. The results provide additional evidence of non-inferiority of 3 versus 6 months of treatment, mainly in patients under CAPOX. On the other hand, an increase on the rate of patients with detectable CTCs and of the absolute CTC number was shown in patients under FOLFOX, for 6 months. Microbial DNA fragments that are possibly originated from the intestine, in the blood of stage III CRC patients is significantly correlated with the detection of CTCs, at baseline, as well as with the increase in the number of CTC, during the first three months of treatment administration. Finally, subsequent studies are needed to further evaluate possible resistance mechanisms and emergence of resistant clones, in stage III patients under oxaliplatin-based treatments.

## Figures and Tables

**Table 1:** Total number of patients, gender, chosen treatments and number of patients at each time point.

Characteristics	Frequency (*n* = 121)	%
Age (range)	62 (37–83)	
<70	83	68.9
≥70	38	31.4
Gender		
Male	73	60.3
Female	48	39.7
PS (ECOG)		
0–1	120	99.2
≥2	1	0.8
Surgery		
Yes	121	100
No	0	0.0
Location		
Colon/Sigmoid	112	92.6
Rectum	9	7.4
Right/Left site		
Right colon	28	23.1
Left colon	93	76.9
Histology		
Adenocarcinoma	99	85.3
Mucinous	17	14.7
Unknown	5	
Regimen		
FOLFOX	50	41.3
CAPOX	71	58.7
Treatment Duration		
3 months	37	30.6
6 months	84	69.4
Regimen and Duration		
FOLFOX—3 months	9	7.4
FOLFOX—6 months	41	33.9
CAPOX—3 months	28	23.1
CAPOX—6 months	43	35.6

**Table 2:** Number of CTCs, at all three different time points, during treatment.

Timepoint	Immunofluorescence (IFAT)			RT-qPCR	
	Pos/Neg	No	Median CTC No (Range)	No (%)	Median Copy Number (Range)
**Baseline**	Pos	55 (45%)	3 (1–119)	46 (38.3%) *	1.1 (0.71–6.7)
	Neg	66 (55%)		74 (61.7%)	
**3 months**	Pos	31 (44.9%)	1 (1–6) *	24 (34.8%) *	1.1 (0.75–2.4)
	Neg	38 (55.1%)		45 (65.2%)	
**6 months**	Pos	24 (44.4%)	2 (1–134) *	23 (42.6%) *	1.2 (0.71–12.33)
	Neg	30 (55.6%)		31 (57.4%)	

* *p* < 0.001.

**Table 3:** Changes in the detection and number of CTCs at during chemotherapy.

**Detection of CTCs**	**Baseline—3 Months**	**Baseline—6 Months**	**3 months—6 Months**
**+/+**	27.5%	22.2%	57.1%
**+/-**	20.3% *	5.6%	14.3%
**-/-**	33.3%	50%	14.3%
**-/+**	18.8%	22.2%	14.3%
**Absolute No of CTCs**	**Baseline—3 months**	**Baseline—6 months**	**3 months—6 months**
**Increased**	23.2%	29.6%	42.9% **
**Decreased**	37.7%	14.8%	23.8%
**Stable**	39.1%	55.6%	33.3%

* *p* = 0.044; ** *p* = 0.007.

**Table 4:** Comparison of absolute number of CTCs at each time point and chemotherapy regimen.

	Baseline vs. 3 Months			Baseline vs. 6 Months			3 Months vs. 6 Months		
	Increase	Decrease	*p*	Increase	Decrease	*p*	Increase	Decrease	*p*
**FOLFOX**	7 (16.7%)	9 (21.4%)	0.554	13 (54.2%)	1 (4.2%)	0.001	8 (57.1%)	2 (14.3%)	0.05
**CAPOX**	9 (21.4%)	17 (40.5%)		3 (12.5%)	7 (29.2%)		1 (7.1%)	2(21.4%)	

**Table 5:** Association of microbial DNA detection at baseline with the detection of CTCs during treatment.

		**CTCs Detection (All Treatments)**					
		**Baseline**	**3 Months**	**6 Months**	**Increase between Baseline—3 Months**	**Increase between Baseline—6 Months**	**Increase between 3–6 Months**
	**16S rRNA**	*p* = 0.026	*p* = 0.108	*p* = 0.799	*p* = 0.02	*p* = 0.108	*p* = 1
	***E. coli***	*p* = 0.02	*p* = 0.921	*p* = 0.45	*p* = 0.462	*p* = 0.268	*p* = 0.789
	***B. fragilis***	*p* < 0.001	*p* = 0.160	*p* = 0.887	*p* = 0.036	*p* = 0.163	*p* = 0.574
**Microbial DNA**	***C. albicans***	*p* = 0.029	*p* = 0.392	*p* = 0.257	*p* = 0.201	*p* = 0.11	*p* = 0.5
		**CTCs Detection (FOLFOX only)**					
		**Baseline**	**3 months**	**6 months**	**Increase between Baseline—3 months**	**Increase between Baseline—6 months**	**Increase between 3–6 months**
	**16S rRNA**	*p* = 0.18	*p* = 0.279	*p* = 0.341	*p* = 0.034	*p* = 0.409	*p* = 1
	***E. coli***	*p* = 0.036	*p* = 0.545	*p* = 0.295	*p* = 0.538	*p* = 0.469	*p* = 1
	***B. fragilis***	*p* = 0.01	*p* = 0.643	*p* = 0.326	*p* = 0508	*p* = 0.404	*p* = 0.667
**Microbial DNA**	***C. albicans***	*p* = 0.392	*p* = 0.383	*p* = 0.659	*p* = 0.404	*p* = 0.509	*p* = 0.255
		**CTCs Detection (CAPOX only)**					
		**Baseline**	**3 months**	**6 months**	**Increase between Baseline—3 months**	**Increase between Baseline—6 months**	**Increase between 3–6 months**
	**16S rRNA**	*p* = 0.137	*p* = 0.043	*p* = 0.392	*p* = 0.009	*p* = 0.306	*p* = 0.405
	***E. coli***	*p* = 0.355	*p* = 0.384	*p* = 1	*p* = 0.293	*p* = 0.236	*p* = 0.405
	***B. fragilis***	*p* = 0.013	*p* = 0.013	*p* = 1	*p* = 0.015	*p* = 0.464	*p* = 0.167
**Microbial DNA**	***C. albicans***	*p* = 0.029	*p* = 0.082	*p* = 0.76	*p* = 0.322	*p* = 0.094	*p* = 0.167

## Data Availability

The data presented in this study are available as a Supplementary Materials here, Table S1.

## Supplementary Materials

The following are available online at https://www.mdpi.com/article/10.3390/cancers13143552/s1, Table S1: Raw data material.
